# A fibrin targeted molecular imaging evaluation of microvascular no‐reflow in acute ischemic stroke

**DOI:** 10.1002/brb3.2474

**Published:** 2022-01-13

**Authors:** Xi Chen, Jing Wang, Liang Ge, Gang Lu, Hailin Wan, Yeqing Jiang, Zhenwei Yao, Gang Deng, Xiaolong Zhang

**Affiliations:** ^1^ Department of Radiology, Huashan Hospital Fudan University Shanghai China; ^2^ Department of Intervention and Vascular Surgery, Zhongda Hospital Southeast University Nanjing China

**Keywords:** acute ischemic stroke, fibrin deposition, microvascular no‐reflow, molecular imaging, thrombolysis

## Abstract

**Objective:**

To investigate the relationship between fibrin deposition and “no‐reflow” within microcirculation after thrombolysis in acute ischemic stroke (AIS).

**Materials and methods:**

Experiments were approved by the institutional animal care and use committee. An experimental AIS model was induced in C57BL/6 mice by middle cerebral artery occlusion (MCAO) via the photothrombotic method. Mice were randomly assigned to non‐thrombolytic or thrombolytic treated groups (n = 12 per group). The modified Neurological Severity Score and Fast Beam Balance Test were performed by a researcher blinded to the treatment method. MRI was utilized to evaluate all of the mice. An FXIIIa‐targeted probe was applied to detect fibrin deposition in acute ischemic brain regions by fluorescence imaging. Necrosis and pathological changes of brain tissue were estimated via Hematoxylin and eosin staining while fibrin deposition was observed by immunohistochemistry.

**Results:**

Thrombolytic therapy improved AIS clinical symptoms. The infarct area of non‐thrombolytic treated mice was significantly greater than that of the thrombolytic treated mice (*p* < .0001). Fluorescent imaging indicated fibrin deposition in ischemic brain tissue in both groups, with less fibrin in non‐thrombolytic treated mice than thrombolytic treated mice, though the difference was not significant. Brain cells with abnormal morphology, necrosis, and liquefication were observed in the infarcted area for both groups. Clotted red blood cells (RBCs) and fibrin build‐up in capillaries were found near the ischemic area in both non‐thrombolytic and thrombolytic treated groups of mice.

**Conclusion:**

Fibrin deposition and stacked RBCs contribute to microcirculation no‐reflow in AIS after thrombolytic therapy.

## 1 INTRODUCTION

Acute ischemic stroke (AIS) is one of the most common causes of death and disability worldwide. (Virani et al., 2020) Thrombolytic therapy is widely used to recanalize occluded cerebral blood vessels to save ischemic brain tissue thereby reducing the mortality rate. (Lo, 2008; Lo et al., 2003) Alteplase, which degrades thrombus fibrin, is the only FDA‐approved pharmacotherapy for AIS (Liu et al., 2017; Rha & Saver, 2007). Recanalization of fibrin occluded vessels re‐perfuses the ischemic brain region with blood and oxygen, thus promoting stroke rehabilitation (Huang et al., 2015; Panni et al., 2019). However, accumulating evidence indicates that vessel recanalization from thrombolysis does not always lead to brain reperfusion, possibly attributable to the failure of microvascular reperfusion, a phenomenon termed “no‐reflow” in AIS (Dalkara & Arsava, 2012; El Amki et al., 2020; Lee et al., 2016). The underlying mechanism of AIS “no‐reflow” has not yet been fully understood.

Thrombus location, size, and composition are important factors affecting thrombolytic treatment outcome (Friedrich et al., 2015; Liebeskind et al., 2011; Morales‐Vidal & Biller, 2013; Riedel et al., 2011; Rohan et al., 2014). Thrombi causing AIS mainly consist of activated platelets, red blood cells (RBCs), and fibrin (Duffy et al., 2017; Niesten et al., 2014), classifiable into fibrin‐rich and RBC‐rich clots (Jolugbo & Ariëns, 2021). Fibrin forms the basic framework of thrombus and interacts differently with RBCs and platelets. Understanding the relationship between fibrin deposition and “no‐reflow” within microcirculation after thrombolysis can inform AIS thrombolytic therapy, and also help develop new treatments targeting microvascular “no‐reflow.” We hypothesize that fibrin deposition in capillaries is an important mediator of “no‐reflow” leading to insufficient brain recovery.

Upon activation, blood coagulation factor XIII (FXIII), is a key mediator of thrombosis and fibrinolytic resistance (Bagoly et al., 2012; Jaffer et al., 2004; Jaffer et al., 2004; Kohji Kasahara et al., 2010; Kim et al., 2016). In this study, an FXIIIa‐targeted probe was used to track fibrin deposition via near‐infrared fluorescent (NIRF) imaging in an experimental stroke model of a middle cerebral artery occlusion (MCAO) treated thrombolytically.

## 2 MATERIALS AND METHODS

### 2.1 Stroke model creation and treatment

All animal studies were approved by the Institutional Animal Care and Use Committee and performed in accordance with the guidelines for the care and use of laboratory animals. An AIS model was induced in a total of 48 male C57BL/6J mice, aged 10–12 weeks and weighing between 22 and 28 g (Shanghai Laboratory Animal Center, Chinese Academy of Science, China). Animals were habituated for 1 week under standard conditions with adequate food and fresh water under normal circadian rhythm prior to experimentation. Right‐hemisphere focal cerebral ischemia was induced by occlusion of a proximal branch of the middle cerebral artery (MCA) via the photo‐thrombotic method (Cotrina et al., 2017). Briefly, a photosensitive Rose Bengal dye (0.1 mL of 10 g/L each mouse) was administered intravenously, after which a laser beam (35.5 Candela) was applied for approximately 5 min through the skull base to irradiate the MCA until blood flow stopped. Mice were randomly assigned to thrombolytic or non‐thrombolytic treatment groups, with 12 mice per group and each group was divided into two batches—one for weighing, scoring, behavior test survival analysis, and the other for MRI, NIRF imaging, and pathological analysis. Six sham mice were treated with identical steps sans laser irradiation. Mice in the thrombolytic group were treated with rt‐PA (1 mg/mL, 10 mg/Kg, Boehringer‐Ingelheim Co. Ltd.) by slow infusion via the tail vein within 1 h after induction. (Su et al., 2008) After intravenous injection of 100 μl rt‐PA, the remainder was continuously administered into the tail vein through a microsyringe pump at a rate of 1000 μl/hr. For mice in the non‐thrombolytic treated group, an equivalent amount of saline was administered in a like manner.

### 2.2 Severity scoring and fast beam balance test

The modified Neurological Severity Score (mNSS) (0–12) was recorded at days 0, 1, 3, and 7 for sham induction, non‐thrombolytic, and thrombolytic treated groups of mice by a researcher unaware of the treatment method. Higher scores indicate more severe symptoms (normal score 0, maximal deficit score 12; Table 1 in supplemental information; Chen et al., 2016). Weights were recorded daily by a blind researcher. Fast Beam Balance Test was performed on Day 1 for sham induction, non‐thrombolytic, and thrombolytic treated mice groups. Prior to the experiment, mice were trained twice a day for 1 week and then deprived of food and water for 24 hr. Mice were placed at one end of the balance beam and encouraged to cross to the other side to obtain food and water, with time and speed recorded and compared between groups.

**TABLE 1:** Modified neurologic severity scores (mNSS)

Motor tests	Points
*Raising the mouse by the tail* 1 Flexion of forelimb 1 Flexion of hindlimb 1 Head moved more than 10° to the vertical axis within 30 seconds	3
*Walking on the floor (normal = 0; maximum = 3)* 0 Normal walk 1 Inability to walk straight 2 Circling toward the paretic side 3 Falling down to the paretic side	3
*Beam balance tests (normal = 0; maximum = 6)* 1 Grasps side of beam 2 Hugs the beam and one limb falls down from the beam 3 Hugs the beam and two limbs fall down from the beam, or spins on beam (> 30 seconds) 4 Attempts to balance on the beam but falls off (>20 s) 5 Attempts to balance on the beam but falls off (>10 s)	6
6 Falls off: No attempt to balance or hang on to the beam (<10 s)	12
Maximum points	

### 2.3 MRI and postprocessing

Cerebral MRI was performed on each rodent via a 7.0‐T MR imaging unit with a mouse four‐channel head surface coil (Bruker, Pharmascan 7.0‐T, Billerica, MA). T2‐weighted imaging (T2WI), while three‐dimensional Fast Low Angle Shot Magnetic Resonance Angiography (3D FLASH‐MRA) images were collected 24 hr after modeling. Imaging parameters for T2WI were: TR = 3000 ms, TE = 40 ms, field of view 18 × 18 mm, matrix 256 × 256. Imaging parameters for 3D FLASH‐MRA: TR = 15 ms, TE = 2.5 ms, field of view 20 × 20 mm, matrix 256 × 256. All sequences were acquired with a slice thickness of 1 mm. Data acquisition and image processing were performed using a Paravision 5.0 software platform (Bruker Biospin, Ettlingen, Germany). 3D cerebrovascular images from 3D FLASH‐MRA were automatically generated and reconstructed on the platform. Image J software (NIH) was employed to measure the infarction size on T2W images for the different groups of mice.

### 2.4 A15 synthesis and NIRF imaging

The FXIIIa‐targeted probe, A15, was synthesized according to the previous study with slight modification (Tung et al., 2003). Briefly, Ac‐GN_13_QEQVSPLTLLK_24_WC, an FXIIIa affinity peptide based on α‐2‐antiplasmin, was compounded by solid‐phase peptide synthesis (purchased from Fubaike Co., Beijing), and then marked with Cy7 (Molecular Probes, Fluorescence, China) via its cysteine side chain (A15‐Cy7). Fluorescent images were obtained with the IVIS Spectrum In Vivo Imaging System (PerkinElmer, USA) at 24 hr post‐injection of A15‐Cy7 (0.5 mg/kg), which was administered immediately after MCAO. Two filter sets (excitation: 743 nm; emission: 767 nm) were used to detect probe‐specific fluorescence (from A15‐Cy7) and auto‐fluorescence (primarily from the skin and other organs). Fluorescent images were then analyzed based on their spectral patterns using Living Imaging Software (PerkinElmer, USA). Results were analyzed by a radiologist who was blinded to the specific treatment method. For analysis of fibrin deposition, regions of interest (ROIs) were selected manually on both hemispheres of the NIRF images. Target‐to‐background ratios (TBRs), defined as Intensity_ROI of ipsilateral ischemic brain_/Intensity_ROI of contralateral normal brain_, were used to quantify fibrin deposition (Klohs et al., 2008).

### 2.5 Histopathological analysis

After MR and NIRF imaging on Day 1, mice brains were harvested after being perfused with 20 ml ice‐cold PBS transcardially, and cut into 4 μm‐thick sections. The degree of necrosis and pathological changes of brain tissue was estimated via hematoxylin and eosin (H&E) staining. Fibrin deposition was observed by immunohistochemistry. Sections were incubated with a primary antibody of anti‐Fibrinogen (Sigma Co.) diluted in PBS solution (1:50) at 4°C overnight. The next day, sections were labeled with a secondary antibody of horseradish peroxidase (Goat anti‐Rabbit IgG (H+L), diluted with PBS 1:1000, Sigma Co.) for 30 min at room temperature followed by staining with diaminobenzidine. Sections were visualized on an inverted fluorescent microscope (Optika, XDS‐3FL4). Morphological analysis and quantification of positive regions were conducted by a certified pathologist.

### 2.6 Statistical analysis

In order to avoid the influence of subjective factors, a completely randomized design was adopted, with all measurements performed blindly with regard to the treatment method. One‐way ANOVA test was used to compare the results of the Fast Beam Balance Test, mNSS scoring, infarct size on T2W images, and TBRs from NIRF images between the non‐thrombolytic and thrombolytic treated groups. Log‐rank (Mantel‐Cox) test was used to compare the survival curve differences between each group. Results were reported as mean ± standard deviation (SD). GraphPad Prism (v.6, GraphPad Software) was used for statistical analysis, while *p* < .05 was considered statistically significant.

## 3 RESULTS

### 3.1 Thrombolytic therapy improves AIS symptoms

**FIGURE 1:**
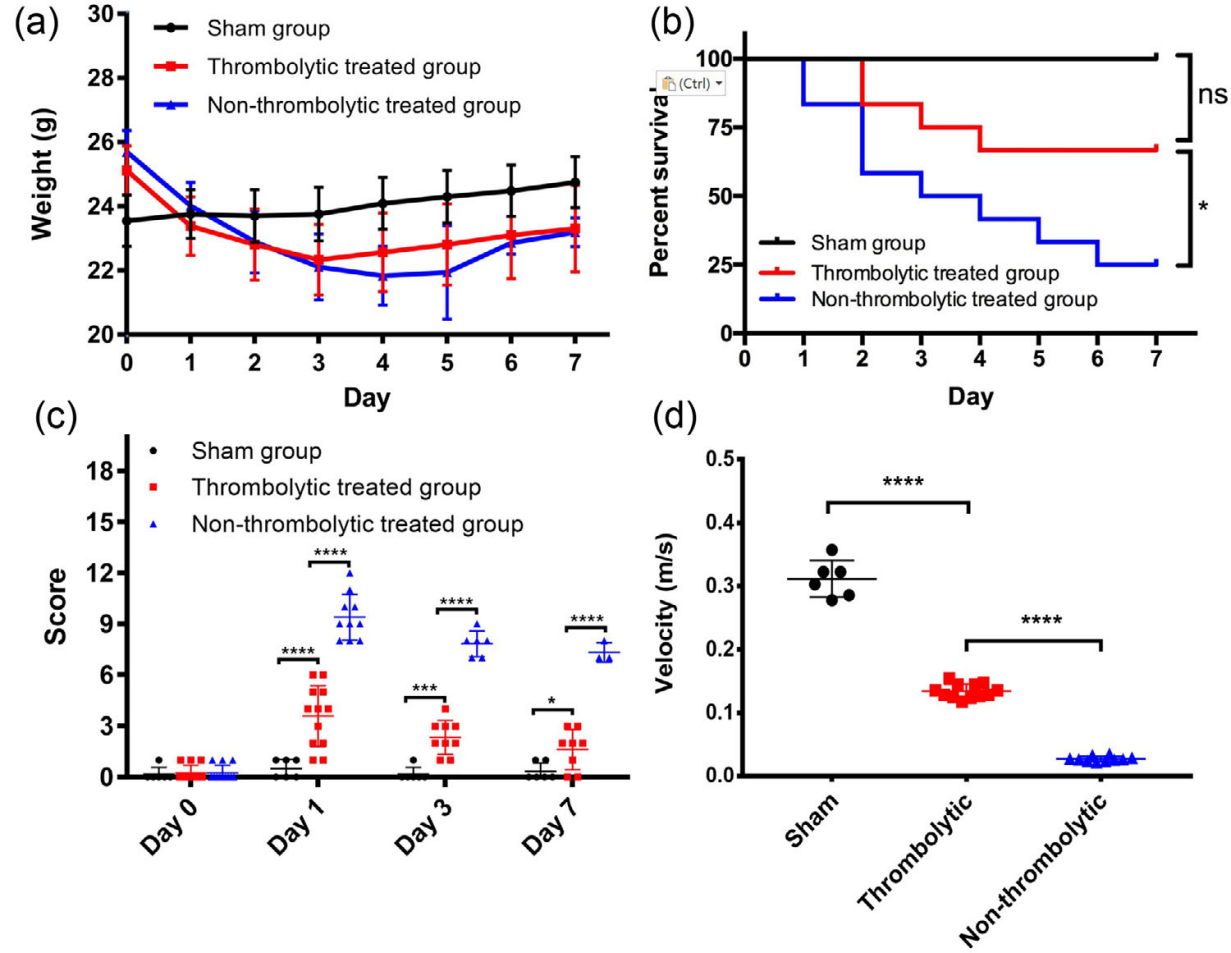
Thrombolytic therapy improves AIS clinical symptoms. (a) Daily weight of mice in sham group (n = 6), thrombolytic treated group (n = 12), and non‐thrombolytic treated group (n = 12). (b) Survival plot of sham induction mice, thrombolytic treated mice, and non‐thrombolytic treated mice with survival rates of 100%, 67%, and 25%, respectively (*p *= .044 between thrombolytic and non‐thrombolytic treated mice, *p* = .1207 between sham induction and thrombolytic treated mice). (c) The mNSS scores of the sham group, thrombolytic treated group, and non‐thrombolytic treated group of mice on days 0, 1, 3, and 7 (thrombolytic *vs* non‐thrombolytic treated groups: *p* < .0001 on all test days; sham vs. thrombolytic treated groups: *p* < .0001 on day 1, *p* = .0002 on day 3, *p* = .0455 on day 7). (D) Result of fast beam balance test on day 1 (*p* < .0001)

Photo‐thrombosis can induce vascular endothelial damage and plaque formation in a targeted MCA, thus causing AIS via cerebral thrombosis. Thrombolytic treatment with rt‐PA at 1 hr after symptom onset resulted in less weight loss (Figure 1a) and significantly improved survival: 67% (8/12) of the thrombolytic treated mice survived, while only 25% (3/12) survived in the non‐thrombolytic group (Figure 1b, *p *= .044 between thrombolytic treated and non‐thrombolytic treated groups, *p* = .1207 between sham induction and thrombolytic treated groups). The mNSS provides a reliable assessment for clinical severity, highly correlated with infarct volume in AIS (Bieber et al., 2019). AIS mice treated with thrombolytic therapy showed significantly improved clinical symptoms compared with non‐thrombolytic treated mice on days 1, 3, and 7 (*p* < .0001 for mNSS on all test days, Figure 1c). After thrombolysis, AIS mice still showed severe clinical symptoms, which gradually recovered as represented by lower mNSS scores compared with sham induction mice (*p* < .0001 on day 1, *p* = .0002 on day 3, *p* = .0455 on day 7, Figure 1c). AIS mice with thrombolytic therapy showed significantly faster speeds than mice without thrombolytic therapy on the Fast Beam Balance Test on day 1 (*p* < .0001, Figure 1d). These findings reveal that thrombolytic therapy with rt‐PA improves clinical symptoms and survival rate for photothrombotic AIS mice.

### 3.2 Thrombolytic therapy recanalizes occluded vessels and reduces infarct area

**FIGURE 2:**
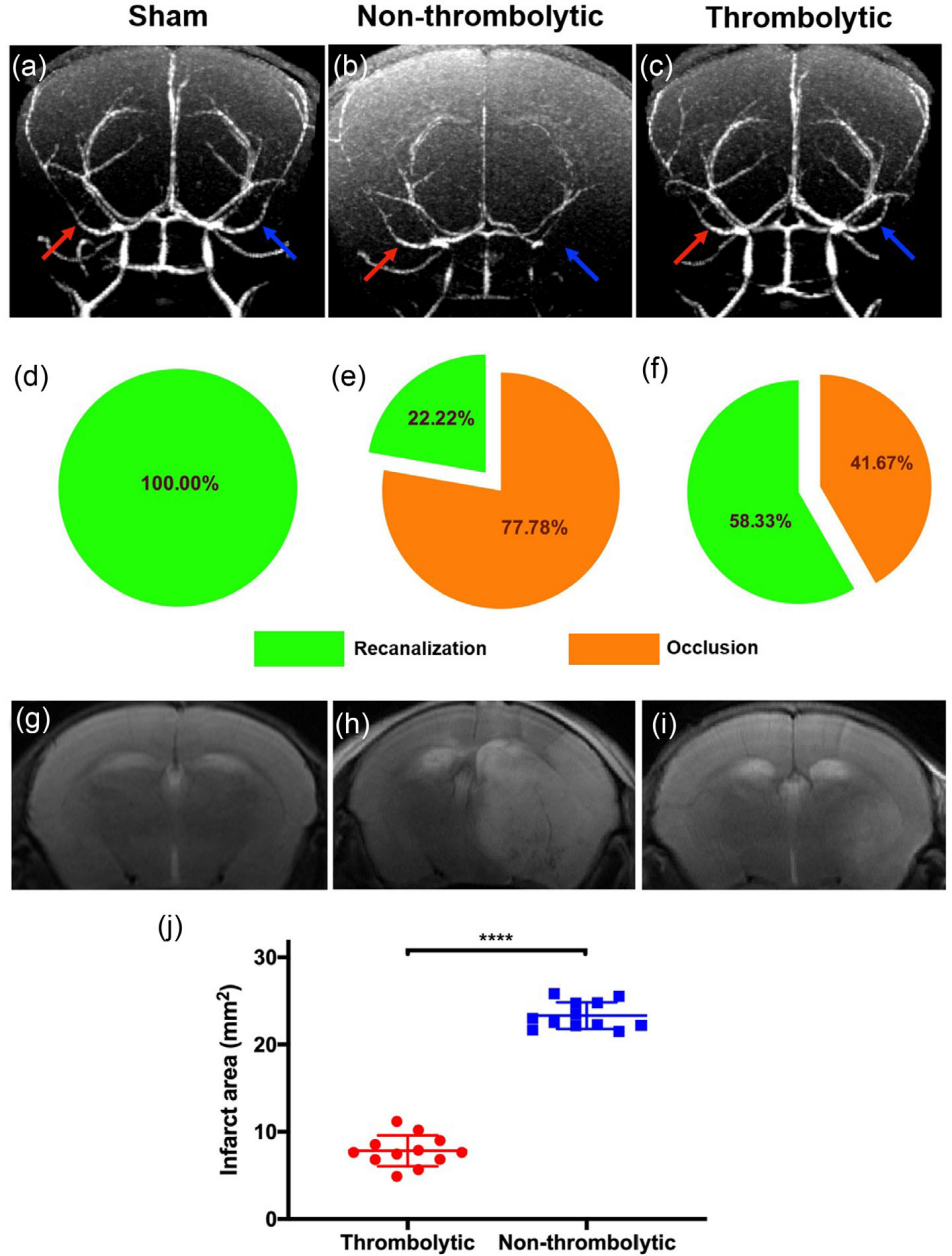
Thrombolytic therapy recanalizes occluded vessels and reduces the infarct area. 3D FLASH‐MRA images of sham induction mice (a), AIS mice in non‐thrombolytic treated (b), and thrombolytic treated (c) groups, with red arrows indicating contralateral MCA and blue arrows indicating ipsilateral MCA. Patency rate of the right MCA in sham induction group (d), non‐thrombolytic treated group (e), and thrombolytic treated group (f). Brain T2W MR images of sham induction mice (g), AIS mice in non‐thrombolytic treated (h), and thrombolytic treated (i) groups. (j) Infarct lesion area was measured (n = 12) revealing that thrombolytic treated mice experienced significantly smaller lesion areas than non‐thrombolytic treated mice (*p* < .0001)

The presence of AIS and the effect of thrombolytic therapy on Day 1 after modeling was determined via MRI. 3D FLASH‐MRA images were obtained for sham induction mice, AIS mice in the thrombolytic and non‐thrombolytic treated groups. Bilateral MCAs were delineated clearly in sham induction mice (Figure 2a, red and blue arrow). For AIS mice in the non‐thrombolytic treated group, the contralateral MCA was still well displayed (Figure 2b, red arrow), while the ipsilateral MCA was obstructed and not visible (Figure 2b, blue arrow), indicating the successful creation of an AIS model. For mice in the thrombolytic treated group, the ipsilateral MCA signal on 3D FLASH‐MRA (Figure 2c) was recovered, which demonstrated the recanalization of the occluded MCA. Twenty‐four hours after modeling, the right MCA was 100% patent for the sham induction mice, 22.22% for the non‐thrombolytic treated group, and 58.33% for the thrombolytic treated group, as calculated from 3D FLASH‐MRA images (Figure 2d–f). T2W images after 24 hr of modeling showed that the infarct area of AIS mice with thrombolytic therapy (7.40 ± 1.46 mm^2^) was significantly smaller than that in mice without thrombolysis (23.39 ± 1.58 mm^2^; *p* < .0001, Figure 2h–j). No abnormal signal was found in the brain of sham induction mice (Figure 2g). These results indicate that thrombolytic therapy can effectively recanalize the occluded MCA thus greatly reducing the infarction area. However, thrombolysis even at 1 hr after stroke occurrence cannot completely avoid infarction despite successful rapid recanalization.

### 3.3 NIRF imaging uncovers fibrin deposition after thrombolysis

**FIGURE 3:**
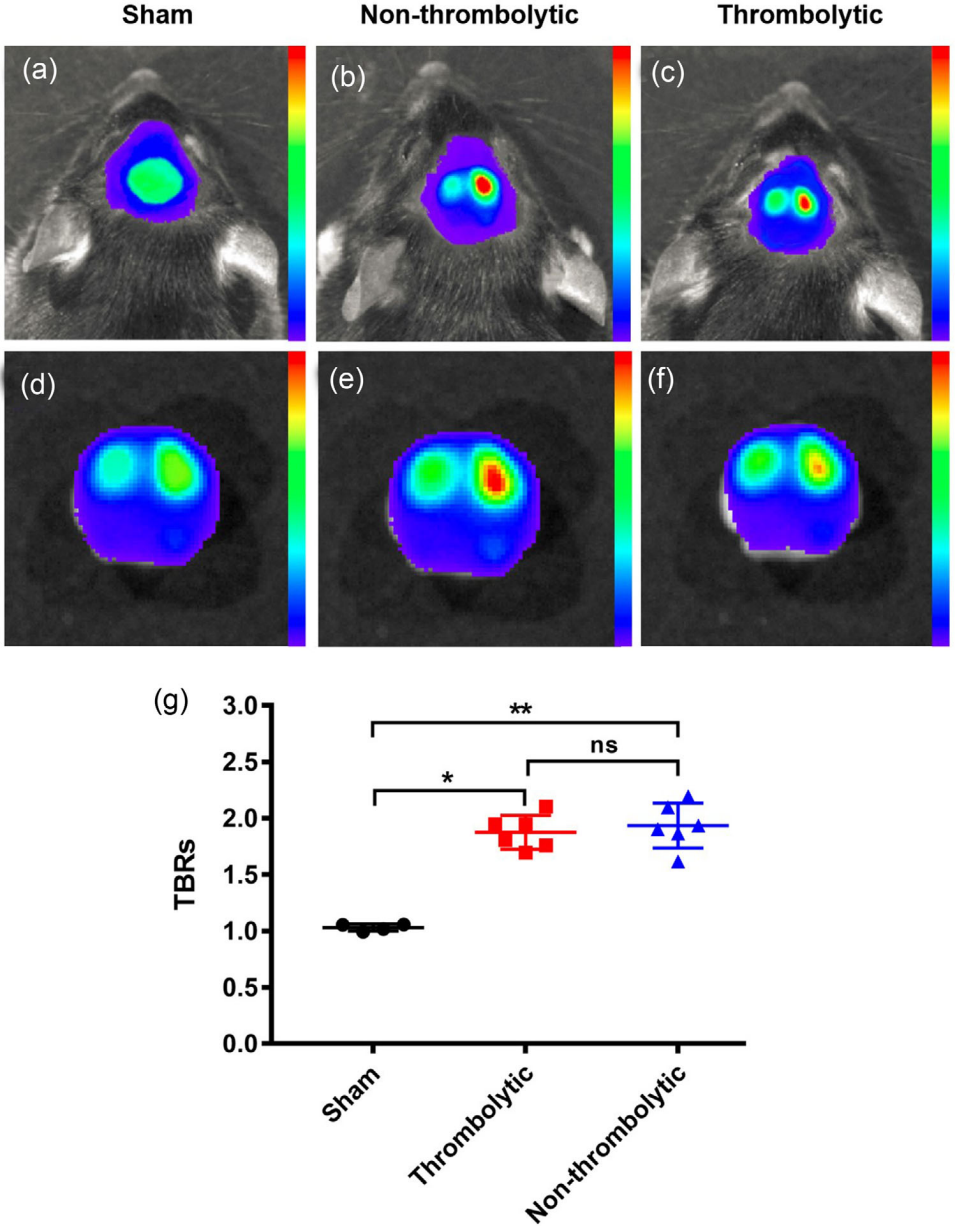
NIRF imaging uncovers fibrin deposition after thrombolysis. In vivo (a‐c) and in vitro (d‐f) NIRF images of sham induction mice (a, d), AIS mice in non‐thrombolytic treated (b, e), and thrombolytic treated (c, f) groups. (g) Significant differences in fibrin deposition were found in thrombolytic treated (*p* = .013) and non‐thrombolytic treated (*p* = .0029) mice compared with sham induction mice. No significant difference in TBRs was found between non‐thrombolytic treated group and thrombolytic treated group of mice (*p* = .644)

In order to find out what hindered brain recovery after thrombolysis, an FXIIIa‐targeted probe (A15‐Cy7) to track fibrin deposition in AIS via NIRF imaging, as previously reported (Jaffer et al., 2004; Kim et al., 2016; Tung et al., 2003) The sham induction mice showed low fluorescent intensity in bilateral brain regions both in vivo (Figure 3a) and ex vivo (Figure 3d). For mice in the non‐thrombolytic treated group (Figure 3b,e) and thrombolytic treated group (Figure 3c,f), high fluorescence intensity was displayed in the affected side with relatively low intensity on the contralateral side. Significant fibrin deposition was found in AIS mice both with and without rt‐PA thrombolysis compared with sham induction mice (*p* = .013 for thrombolytic treated mice, *p* = .0029 for non‐thrombolytic treated mice). No significant difference in TBRs was found between the non‐thrombolytic treated group and the thrombolytic treated group of mice (*p* = .644) though TBR in the thrombolytic treated group was slightly lower (Figure 3g), indicating fibrin deposition in ipsilateral brain tissue even after successful thrombolytic therapy.

### 3.4 Histopathological analysis reveals RBC aggregation and fibrin deposition in capillaries

**FIGURE 4:**
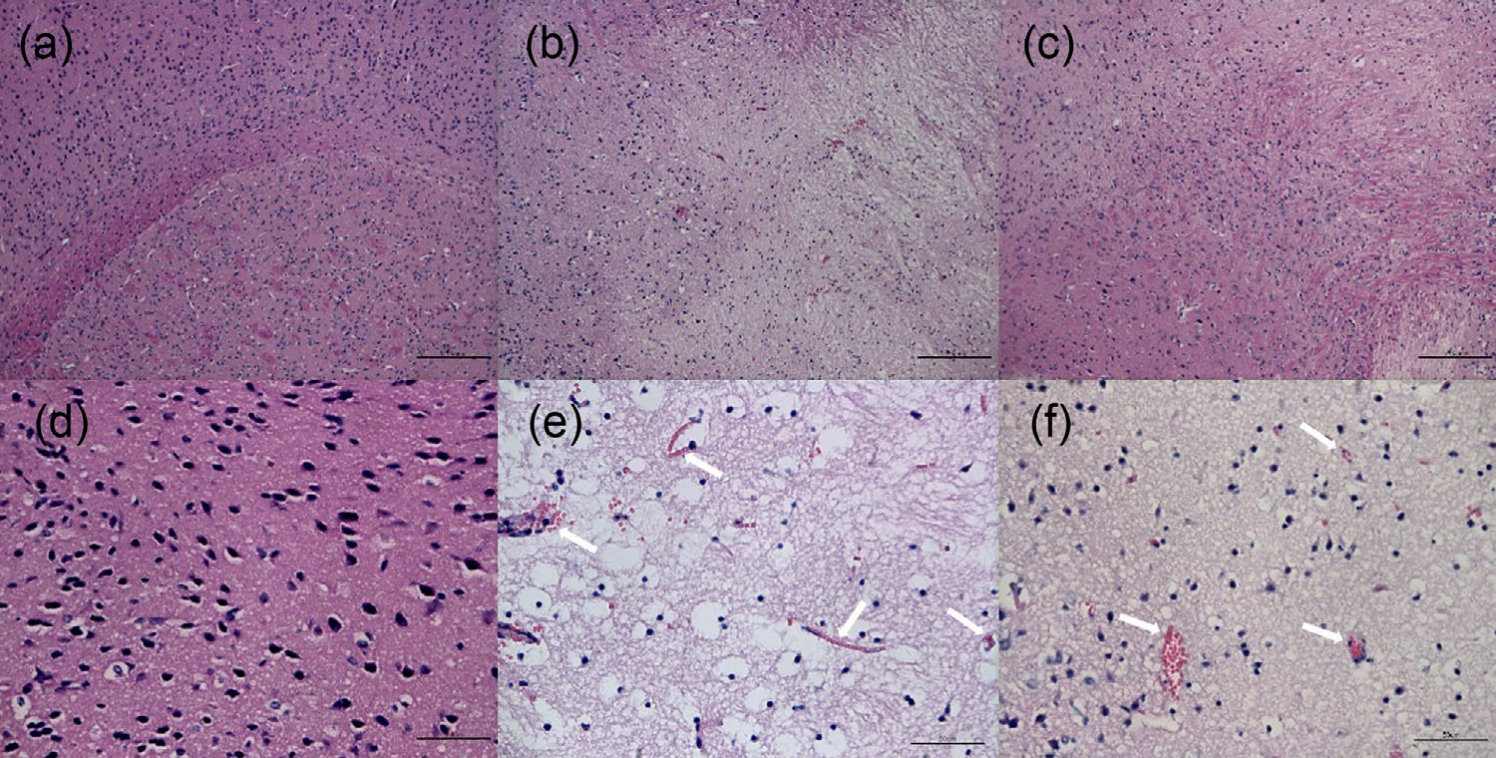
H&E staining of contralateral normal brain (a and d), non‐thrombolytic treated mice (b and e) and thrombolytic treated mice (c and f) with different magnification (a–c 10×, scale bar = 200 μm; d–f 40×, scale bar = 50 μm). Brain cells maintained normal contralateral morphology in normal unimpacted brain regions (a and d). Necrotic tissue area in non‐thrombolytic treated mice (b) was substantially larger than in thrombolytic treated mice (c). Demarcation between necrotic tissue and normal tissue can be seen in both groups (b and c). (f) Accumulated RBCs in micrangiums and capillaries were found in affected brain regions in thrombolytic treated mice

**FIGURE 5:**
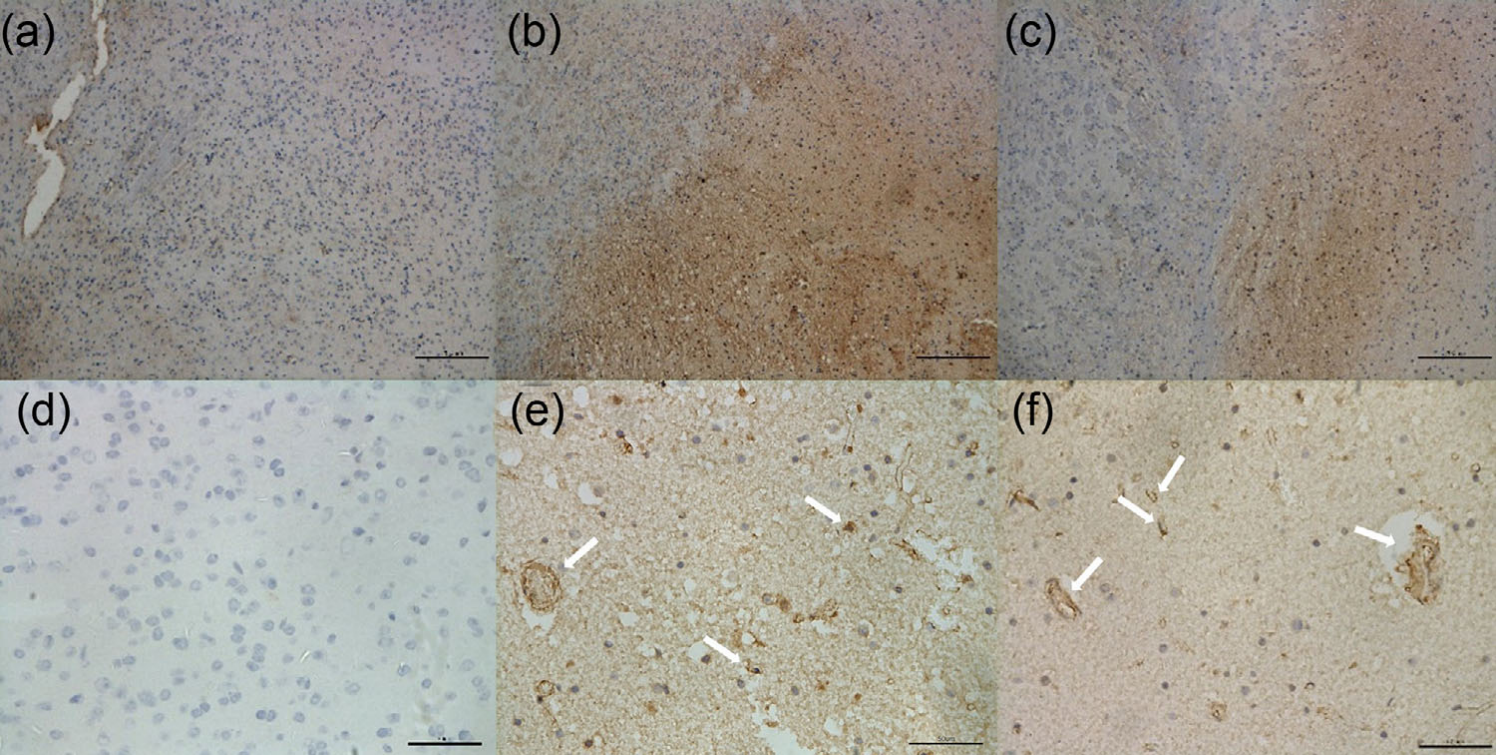
Immunohistochemistry staining of contralateral normal brain (a and d), non‐thrombolytic treated mice (b and e) and thrombolytic treated mice (c and f) with different magnification (a–c 10×, scale bar = 200 μm; d–f 40×, scale bar = 50 μm). Limited positive staining of fibrin was found in contralateral normal brain (a and d). Fibrin deposited area in non‐thrombolytic treated mice (B) was obviously larger than that in thrombolytic treated mice (c). Demarcation between necrotic tissue and normal tissue can be seen in both groups (b and c). (f) Clotty fibrin deposition was observed in micrangiums and capillaries of mice with thrombolytic therapy

After imaging, brains were harvested for histopathological analysis. Contralateral unaffected brain regions maintained normal morphology (Figure 4a,d). The border between the ischemic and non‐ischemic areas was clearly delineated with H&E staining and immunohistochemical staining in both non‐thrombolytic (Figures 4b and 5b) and thrombolytic treated mice (Figures 4c and 5c). Non‐thrombolytic treated mice manifested an obviously larger infarction than thrombolytic treated mice. Brain cell liquefactive necrosis was found in the infarct area of non‐thrombolytic treated mice (Figure 4b,e). An accumulation of RBCs stacked in micrangiums and capillaries was detected at the border of the ischemic area (Figure 4e,f, as indicated by arrows). Immunohistochemistry staining of fibrin was undertaken to detect fibrin deposition in the ischemic brain area. Unimpacted contralateral brain tissue showed sporadic fibrin staining (Figure 5a,d). Patchy fibrin was found in the infarction area in both groups (Figure 5b,c,e,f), which accords with NIRF imaging results. Fibrin deposits occupied a significantly larger area in non‐thrombolytic treated mice (Figure 5b) compared with thrombolytic treated mice (Figure 5c). Interestingly, when comparing the H&E staining (Figure 4c) and fibrin staining of adjacent sections (Figure 5c) in thrombolytic treated mice, the area of fibrin deposition was larger than the area of necrosis, indicating that fibrin deposited not only within the infarction area but also in the penumbral zone. Clotty fibrin deposition was found in micrangium and capillary walls in both thrombolytic and non‐thrombolytic mice (Figure 5e,f, indicated by arrows). Therefore, we conclude that both RBC accumulation and fibrin deposition lead to “no‐reflow” of microcirculation after thrombolytic therapy.

## 4 DISCUSSION

Thrombolytic treatment of AIS with rt‐PA within 4.5 hr after symptom onset has shown beneficial effects for patients, the efficacy of which is particularly time‐dependent, with earlier treatment generating better stroke outcome (Emberson et al., 2014). However, no‐reflow of microcirculation hinders the full recovery of affected brain tissue even with prompt and successful recanalization of occluded vessels (Dalkara & Arsava, 2012). Our study ascertained that despite reopening an obstructed artery as early as 1 hr after inducing a stroke in mice, significant infarction still occurred in the affected brain region. Treatments targeting microvascular “no‐reflow” may therefore result in increased recovery of at‐risk tissue. Duffy et al. reported that recanalization of microcirculation after thrombolytic therapy is one of the key factors determining the functional and morphological recovery of ischemic brain tissue (Duffy et al., 2017). Recovery of microcirculation blood flow should achieve full perfusion of blood in the ischemic hypoxia zone, and restore neurocyte functionality, while improving the effect and prognosis of thrombolytic treatment (Lee et al., 2016). However, the underlying no‐reflow mechanism has not been fully understood. El Amki et al. identified neutrophils plugging brain capillaries hindering microvascular reperfusion in AIS (El Amki et al., 2020), presumably caused by the inflammatory response in the ischemia area. In this study, molecular imaging with a special probe combined with histopathological analysis demonstrated that fibrin deposition contributes to the microcirculation no‐reflow phenomenon inhibiting successful microcirculation recanalization with rt‐PA.

Fibrin serves as the framework for thrombus and affects the fibrinolysis rate. Non‐invasive imaging of fibrin using positron emission tomography, MRI, computed tomography, and NIRF has been widely investigated for thrombotic diseases, allowing for accurate diagnosis while guiding treatment for thrombi at different sites such as coronary vessels, pulmonary artery, deep veins, and cerebral vessels (Andia et al., 2014; Ay et al., 2014; D.‐E. Kim et al., 2016; J.‐Y. Kim et al., 2015; Spuentrup et al., 2005; Tung et al., 2003).

This study utilized a fibrin‐targeted NIRF probe to reveal fibrin accumulation after reopening the occluded artery via in vivo NIRF imaging. A histopathological analysis confirmed that deposited fibrin, as well as accumulated RBCs entangled by fibrin in micrangiums and capillaries around the infarct area, leads to insufficient reperfusion of microcirculation, a condition known as “no‐reflow.” Therefore, the in vivo non‐invasive visualization of deposited fibrin after thrombolysis in AIS with rt‐PA can reflect the degree of reperfusion within the microcirculation and the severity of no‐reflow, providing critical guidance for further clinical treatment and assessment.

## 5 LIMITATIONS

Histopathological findings in this study determined that the no‐reflow phenomenon is caused by fibrin deposits and RBC accumulation in microvessels. Fibrin‐targeted molecular imaging should be capable of evaluating microvascular no‐reflow in acute ischemic stroke; however, there is no direct in vivo evidence supporting this. Future studies can investigate microcirculation blood flow and fibrin deposit quantification.

## 6 CONCLUSION

Molecular imaging of fibrin deposition after recanalization of occluded vessels in AIS can reflect the degree of microcirculation reperfusion while indicating the no‐reflow severity. Post‐thrombolysis in vivo visualization of deposited fibrin offers a potential non‐invasive method to evaluate microcirculation patency thereby providing prompt guidance for next stage clinical treatment.

### PEER REVIEW

The peer review history for this article is available at https://publons.com/publon/10.1002/brb3.2474

## Supporting information

Supporting Information

## Data Availability

The data that support the findings of this study are available from the corresponding author upon reasonable request.
