# Supplementary material for: A fibrin targeted molecular imaging evaluation of microvascular no‐reflow in acute ischemic stroke
Source: Brain Behav. 2022 Jan 13;12(2):e2474. doi: 10.1002/brb3.2474 (PMC8865146; doi:10.1002/brb3.2474)
Supplement: Supplementary file 1 — Supporting Information [file BRB3-12-e2474-s001.docx]

**Table 1**

Modified neurologic severity scores (mNSS)

| Motor tests | Points |
| --- | --- |
| *Raising the mouse by the tail*  1 Flexion of forelimb  1 Flexion of hindlimb  1 Head moved more than 10° to the vertical axis within 30 seconds  *Walking on the floor (normal=0; maximum=3)*  0 Normal walk  1 Inability to walk straight  2 Circling toward the paretic side  3 Falling down to the paretic side  *Beam balance tests (normal=0; maximum=6)*  1 Grasps side of beam  2 Hugs the beam and one limb falls down from the beam  3 Hugs the beam and two limbs fall down from the beam, or spins on beam (>30 seconds)  4 Attempts to balance on the beam but falls off (>20 seconds)  5 Attempts to balance on the beam but falls off (>10 seconds)  6 Falls off: No attempt to balance or hang on to the beam (<10 seconds)  Maximum points | 3  3  6  12 |
